# Acoustic crypsis in communication by North Atlantic right whale mother–calf pairs on the calving grounds

**DOI:** 10.1098/rsbl.2019.0485

**Published:** 2019-10-09

**Authors:** Susan E. Parks, Dana A. Cusano, Sofie M. Van Parijs, Douglas P. Nowacek

**Affiliations:** 1 Department of Biology, Syracuse University, 114 Life Sciences Complex, Syracuse, NY 13244, USA; 2 NOAA Fisheries, Northeast Fisheries Science Center, 166 Water Street, Woods Hole, MA 02543, USA; 3 Nicholas School of the Environment and the Edmund T. Pratt, Jr. School of Engineering, Duke University Marine Lab, 135 Duke Marine Lab Road, Beaufort, NC 28516, USA

**Keywords:** acoustic crypsis, baleen whale, predator avoidance, right whale, eavesdropping

## Abstract

Mammals with dependent young often rely on cryptic behaviour to avoid detection by potential predators. In the mysticetes, large baleen whales, young calves are known to be vulnerable to direct predation from both shark and orca predators; therefore, it is possible that mother–calf pairs may show cryptic behaviours to avoid the attention of predators. Baleen whales primarily communicate through low-frequency acoustic signals, which can travel over long ranges. In this study, we explore the potential for acoustic crypsis, a form of cryptic behaviour to avoid predator detection, in North Atlantic right whale mother–calf pairs. We predicted that mother–calf pairs would either show reduced calling rates, reduced call amplitude or a combination of these behavioural modifications when compared with other demographic groups in the same habitat. Our results show that right whale mother–calf pairs have a strong shift in repertoire usage, significantly reducing the number of higher amplitude, long-distance communication signals they produced when compared with juvenile and pregnant whales in the same habitat. These observations show that right whale mother–calf pairs rely upon acoustic crypsis, potentially to minimize the risk of acoustic eavesdropping by predators.

## 1. Background

In mammals, neonates and juveniles are often subject to higher rates of mortality from predation than mature adults [1,2]. This selective pressure has resulted in a range of behavioural and physical adaptations that improve the survivorship of the vulnerable offspring during their development. Cryptic behaviours to reduce detection by predators have been observed in a range of terrestrial mammals, including visual crypsis and hiding behaviours; reduction in olfactory cues to limit scent detection; and acoustic crypsis to limit eavesdropping or cue detection of the young [3–5].

Many marine species rely on acoustic signals for social communication as these signals can propagate efficiently underwater [6]. These long-range communication signals can be intercepted by predators, putting the signaller at increased risk of predation [7,8]. There is evidence for acoustic crypsis from multiple smaller marine mammal species, which produce signals that are less detectable by their marine mammal predators [9]. Selective pressures to limit detection by predators of mothers with vulnerable young may lead to the modification of acoustic signals, either through acoustic crypsis by producing lower-amplitude signals or through acoustic ‘hiding’ by suppression of social signal production with vulnerable young.

Mysticetes, or baleen whales, have very low adult mortality from predation, with only pods of orca whales (*Orcinus orca*) capable of killing a healthy adult. The predation of young calves is more common, with evidence of predation events by orca and large shark predators in multiple species [10–12]. Mother–calf baleen whale pairs have been hypothesized to produce low-amplitude calls and have lower call rates to avoid detection by other whales or potential predators in the area [13]. Two studies in Australia have shown evidence for acoustic crypsis in mother–calf pairs, including reduced-amplitude sound production by humpback whales (*Megaptera novaeangliae)* [14], and reduced-amplitude sounds with low call rates by Southern right whales (*Eubalaena australis*) [15].

North Atlantic right whales (*Eubalaena glacialis*) are an endangered baleen whale, with approximately 500 individuals left in the entire species [16]. Limited data are available regarding the calling behaviour of baleen whale mother–calf pairs on the calving grounds [17]. In this study, we use acoustic biologging tags to explore whether right whale mother–calf pairs exhibit acoustic crypsis in the form of lower-amplitude signal production or acoustic hiding through reduced acoustic signal production, when the calves are most vulnerable to predation in the first three months after birth.

## 2. Methods

### (a) Acoustic data collection

Data were collected on the calving grounds from juvenile, pregnant and/or lactating North Atlantic right whales in 2006, and 2014–2016 in the waters of the Southeastern United States (SEUS) off the coasts of Georgia and Florida during the peak presence of right whales during the months of January and February. Suction-cup attached archival acoustic recording tags (Dtags) [18] were used to collect acoustic data from individual right whales following methods outlined in Nowacek *et al*. [19]. These tags recorded acoustic, movement and orientation data, including a three-axis accelerometer, magnetometer and pressure sensor. Acoustic data were sampled at either 64 kHz (2006) or 96 kHz (2014–2016). Orientation sensors were sampled at 50 Hz.

### (b) Acoustic analyses

Tag records greater than 20 min in duration were retained for analysis and the first 5 min of data were omitted from analysis to account for any potential behavioural response to tagging [20]. Acoustic recordings were screened for calls using acoustic analysis software Raven Pro 1.5 (Cornell University) and assigned to the tagged whale following methods described in detail in [21]. Detected calls were classified to broad signal categories using the call type characteristics described in [22–24]. These included tonal and broadband calls including *Upcalls, Low calls, High calls, Hybrid calls* and *Gunshots*. A range of shorter duration, broadband amplitude-modulated pulsed signals were identified in the recordings and were grouped into *Pulsed* *calls*, with subsets based on the pairing with tonal calls or the number of discrete pulses in the signal (*Paired grunts* (always produced immediately before a tonal call), *Single pulse*, *Double pulse, Pulsive* (more than two pulses)) [21]. Additional lower-amplitude unstructured calls, consistent with sounds produced by right whale calves [25], were detected on tags attached to lactating females and labelled as *Calf* calls.

Sound clips were made for each whale sound and included 0.5 s of sound before and after each sound for background noise measurements. All sound clips were corrected for a single-pole high-pass filter at 400 Hz on the Dtag using a script from the Dtag MATLAB toolbox [18] and then decimated in MATLAB to a 16 kHz sampling rate. The root-mean-squared (RMS) background noise level was estimated as the minimum value of eight 125 ms segments from the 0.5 s before and after the signal of interest across the full bandwidth. The call received levels (RL_RMS_) (dB re:1 µPa) were calculated by integrating the square of the instantaneous pressure as a function of the time window, defined as a 125 ms window centred on the most energetic part of the call clip using custom scripts in Matlab R2013a (Mathworks, Inc.) and then subtracting the background noise levels from the signal logarithmically. These call received levels represent an apparent source level for these calls to allow for comparison of the relative intensity of different calls within and between tag deployments owing to the positioning of the tag on the back of the whales.

### (c) Statistical analyses

**Figure 1.**
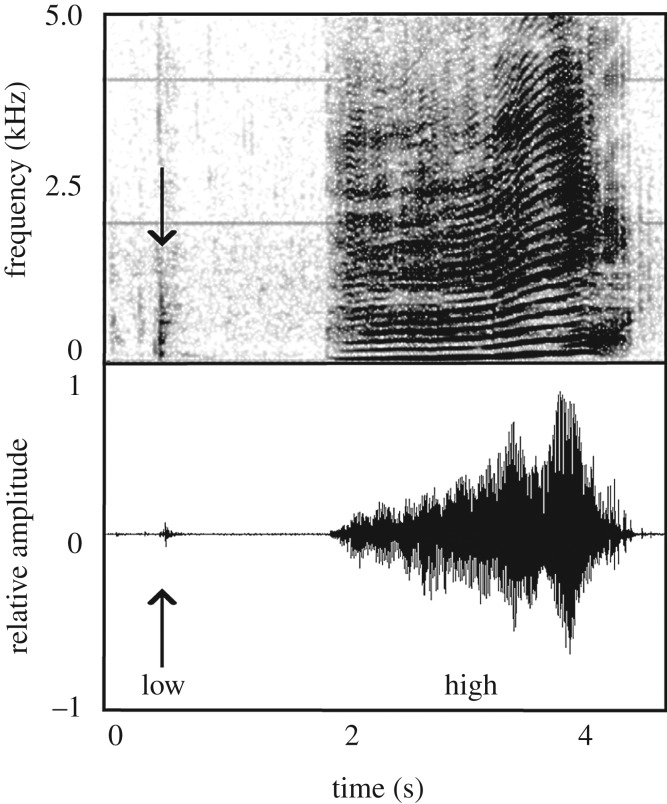
Spectrogram and waveform of an Upcall (high amplitude) and a *Single pulse* (low amplitude) signal produced by a pregnant female on 25 January 2016, highlighting the difference in call amplitude of the signal types. 16 kHz sampling rate, 1024 FFT, Hamming Window, 90% overlap.

All statistical analyses were performed in R [26]. To differentiate between call types that could be detected over short versus longer ranges owing to differences in amplitude, calls were grouped into two call categories based on the results of a *k*-means clustering algorithm implemented in R using the *stats* package [26]. Only calls with a signal-to-noise (SNR) ≥10 dB were included in the *k*-means clustering. This analysis subsequently grouped the calls into high- and low-amplitude based on the standardized RL_RMS_ of the calls (figure 1). The low-amplitude *Paired* calls were omitted from this analysis as they always occurred paired with a higher-amplitude call. The call rate per hour was calculated as the total number of calls in each category detected in an individual tag record of any SNR, divided by the total duration of the audio recording on the tag.

To assess the differences in call type usage (low- versus high-amplitude call classes) between lactating females and other age/sex classes, two methods were used. First, differences in call rates between the two call amplitude classes were assessed using generalized linear mixed models (GLMMs) with a negative binomial distribution, a log offset for time and whale ID as a random effect to account for individuality using the *glmmTMB* package [27]. *Post hoc* analyses were run using the package *emmeans* [28] and contrasts were conducted using Dunnett-style contrasts with the ‘mvt’ method. Next, comparisons were made in the proportion of the repertoire that both call types comprised between lactating females and other age/sex classes. GLMMs were used with binomial error distributions for proportions and whale ID as a random effect. *Post hoc* analyses were run using the ‘mvt’ method. For both models, each tag deployment was considered one observation.

## 3. Results

A total of 16 Dtags were attached to right whales with attachment durations longer than 20 min on the SEUS calving grounds in 2006 (*N* = 4), 2014 (*N* = 4), 2015 (*N* = 1) and 2016 (*N* = 7) for a total of 107.9 h of acoustic data (table 1). These included 11 lactating females with calves and 5 non-lactating whales (2 juvenile males, 1 juvenile female and 2 pregnant females). One individual (ID #3101) was tagged during late pregnancy and then tagged the following month when accompanied by a calf in 2016. A total of 754 calls were detected in the acoustic records. A total of 398 calls with an SNR ≥10 dB and were retained for *k*-means cluster analysis to define high- versus low-amplitude signal classes. Results indicated two clearly defined clusters: calls in cluster 1 were labelled high-amplitude (*n* = 123; mean RL_RMS_ *=* 142 dB re1μPa) and calls in cluster 2 were labelled low-amplitude (*n* = 275; mean RL_RMS_ *=* 122 dB re1μPa). Calls not included in the cluster analysis were assigned to a call amplitude category based on the minimum and maximum RL_RMS_ for each cluster for a total of 159 high-amplitude calls and 595 low-amplitude calls. For the six tags with a minimum of five calls in both the high- and low-amplitude classes, the average within-tag difference in RL for these call classes was 15 ± 2 dB. All 754 calls were subsequently used to identify total and % call types produced by each tagged whale. The full dataset and R script used in the analysis are available in the electronic supplementary material.

**Table 1.** Summary of tag data including: date of tag attachment; whale ID number from the North Atlantic right whale catalogue; state as lactating female (L) or not (N); duration (Dur (h)), tag attachment in hours; total calls (all focal calls detected on the tag record); no. *High calls* (subset of all calls that were high amplitude); no. *Low calls* (subset of calls that were low amplitude); % high-amplitude calls (percentage of calls that were high-amplitude); call rate *High calls* (call rate of high-amplitude calls (calls h^−1^)); call rate *Low calls* (call rate of low-amplitude calls (calls h^−1^).

date	ID	state	Dur (h)	total calls	no. *High calls*	no. *Low calls*	% *High calls*	call rate *High calls*	call rate *Low calls*
21 Feb 2015	3292	L	23.1	116	1	115	0.1	0.04	5.0
22 Feb 2016	3317	L	11.8	73	7	­66	9.6	0.6	5.6
18 Feb 2014	3157	L	11.6	50	2	48	4.0	0.2	4.1
31 Jan 2016	1281	L	6.7	59	12	47	20.3	1.8	7.0
10 Feb 2014	2040	L	5.8	74	8	66	10.8	1.4	11.4
25 Feb 2014	2645	L	5.6	18	0	18	0.0	0.0	3.2
17 Feb 2016	3101	L	4.9	126	16	110	12.7	3.3	22.4
30 Jan 2016	3405	L	4.8	61	4	57	6.6	0.8	11.9
17 Feb 2016	1281	L	2.8	53	8	45	15.1	2.9	16.1
1 Feb 2016	1810	L	1.8	0	0	0	0.0	0.0	0.0
9 Feb 2014	2123	L	1.6	14	13	1	92.9	8.1	0.6
28 Jan 2006	1151	N	18.5	1	1	0	100.0	0.1	0.0
25 Jan 2016	3101	N	5.0	46	45	1	97.8	9.0	0.2
24 Jan 2006	3323	N	1.7	0	0	0	0.0	0.0	0.0
21 Jan 2006	3442	N	1.4	34	32	2	94.1	22.9	1.4
24 Jan 2006	3430	N	0.9	29	10	19	34.5	11.1	21.1

### (a) Call rates

The call rate of low-amplitude calls for lactating females with calves (7.13 ± 2.0 calls h^−1^) was significantly higher than the call rate of high-amplitude calls (0.88 ± 0.70 calls h^−1^, GLMM odds ratio = 0.12 ± 0.06, *t*-ratio = −4.312, *p* = 0.0010). The GLMM revealed no statistically significant differences compared to other age/sex classes; however, non-lactating animals showed a trend of higher call rates for high-amplitude calls (3.21 ± 2.29 calls h^−1^) and lower rates of low-amplitude calls (0.80 ± 1.15 calls h^−1^) compared to lactating females with calves. This result is likely owing to the unbalanced data (more lactating females sampled), small sample size and skewed data owing to a small number of outliers (table 1).

### (b) Call type usage

**Figure 2.**
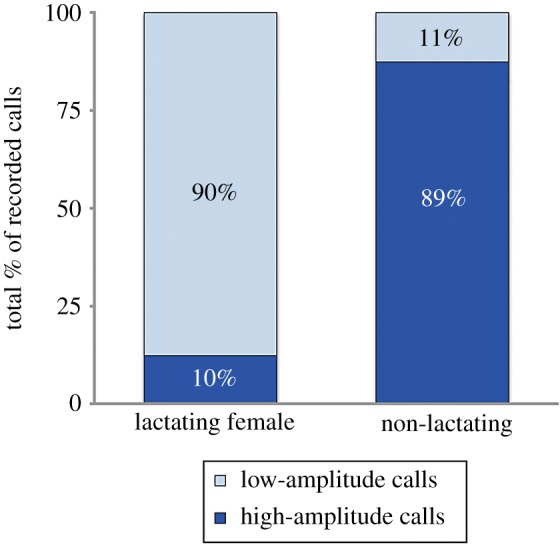
Proportion of high- versus low-amplitude signal production by lactating females and non-lactating whales on the calving grounds. (Online version in colour.)

The results of the GLMM reveal that low-amplitude calls comprise a significantly larger proportion of the call repertoire for lactating females than for non-lactating whales (table 2 and figure 2).

**Table 2.** *emmeans* results from the generalized linear models with the probability of detecting high- and low-amplitude calls in both group compositions and the pairwise contrast (comparison).

call type	lactating females (proportion)	non-lactating animals (proportion)	lactating females versus non-lactating animals (estimate ± s.e.)
low amplitude	0.90 ± 0.05	0.11 ± 0.10	4.36 ± 1.20 *z* ratio = 3.635 *p* = 0.0003
high amplitude	0.10 ± 0.05	0.89 ± 0.08

## 4. Discussion

Acoustic crypsis is a behavioural adaptation to reduce detection by eavesdroppers, including predators. This approach towards reducing conspicuousness is beneficial for species that rely primarily on acoustic signals for communication. Acoustic crypsis may be particularly effective in aquatic environments where the potential range for eavesdropping by predators is greater owing to more efficient signal propagation in water than in air. Right whale mothers produced a higher proportion of quieter, lower-amplitude acoustic signals than pregnant or juvenile whales in the same habitat, suggesting that right whales do use acoustic crypsis when calves are the most vulnerable to predation. This finding is consistent with a previous study of humpback whale and Southern right whale mother–calf communication [14,15] where mothers and their dependent calves produced typical acoustic signals at reduced amplitudes, reducing the potential range for detection. However, rather than simply producing the normal acoustic repertoire at lower amplitudes, North Atlantic right whale mothers with young calves switch their repertoire usage to predominantly produce very quiet call types that are rarely detected in recordings from other demographic groups of right whales.

The lower rates of high-amplitude signals detected from mother–calf pairs may be a behaviour to minimize the potential for eavesdropping by predators or by conspecifics. White sharks (*Carcharodon carcharias*) are commonly sighted on the calving grounds off the Southeastern USA, and have been documented feeding on baleen whales [10] and implicated in the mortality of right whale calves in the habitat where our study occurred [12]. Killer whales have been documented attacking right whales and are commonly sighted on the calving grounds for some Southern right whale populations [11,29]. It is likely that high-amplitude acoustic cues between mother–calf pairs could result in an increased risk of predation from orca predators, and potentially sharks if they are capable of hearing these sounds. These lower-amplitude signals may minimize the risk of detection while still allowing mother–calf communication, albeit over relatively short ranges. The average difference of more than 10 dB in the mean RL between call classes would result in a detection range that is an order of magnitude greater for the high-amplitude versus low-amplitude signals (i.e. a high-amplitude upcall detectable at 1 km would compare to a low-amplitude call detectable at only 100 m), assuming cylindrical spreading in this shallow water habitat.

Alternatively, the lower rate of high-amplitude signal production could reflect differences in the social behaviour of mothers with neonates. Previous studies show increased separation of mother–calf pairs from other conspecifics on the calving grounds in both humpback whales and right whales when compared with other whales, possibly in an attempt to isolate young calves from other conspecifics [30,31]. Given the inter-birth interval of right whales (greater than 3 years) and the estimated gestation period of 11–13 months, females are unlikely to be receptive for mating while nursing a calf [32,33]. Therefore, there may be little benefit of social interactions when the calf is young. North Atlantic right whale mother–calf pairs are rarely sighted in close proximity to other whales on the calving grounds. There were only 17 out of 1361 sightings over a period of 13 years of surveys showing associations between a mother–calf pair and another right whale [34], suggesting that social interactions are infrequent. This behaviour of isolation may reduce the occurrence of social contexts necessitating acoustic communication through higher-amplitude signals. Previous studies of right whale acoustic behaviour on the foraging grounds indicated significant variation in call rates of higher-amplitude call types with behavioural state, with the highest call rates associated with social interactions [23,35]. This may explain much higher reported call rates of high-amplitude signals from large aggregations of southern right whale mother–calf pairs in Brazil, where social interactions are more frequent owing to much higher densities of mother–calf pairs on the calving grounds [36].

Similar to the findings from humpback whales and Southern right whales [14,15], we found mother–calf pairs produced reduced-amplitude signals and low call rates of higher-amplitude signals. In our study, we show additional evidence to support the theory of acoustic crypsis by mother–calf pairs, as these individuals produced significantly fewer high-amplitude signals when compared with juvenile and pregnant whales in the same habitat. Future work from additional populations of baleen whales and other cetacean species is needed to further our understanding of how this low-amplitude signal production benefits mother–calf pairs.

## Supplementary Material

PunctuationSoundClips2016.wav

## Supplementary Material

CallVars.xlsx

## Supplementary Material

CallRate.xlsx

## Supplementary Material

RScript_BiolLett_Parksetal.R

## Supplementary Material

Supplemental Figures

## Data accessibility

Final data tables used in the analysis and statistical analysis R-code from this study are provided in the electronic supplementary material.
