# Supplementary material for: Acoustic crypsis in communication by North Atlantic right whale mother–calf pairs on the calving grounds
Source: Biol Lett. 2019 Oct 9;15(10):20190485. doi: 10.1098/rsbl.2019.0485 (PMC6832179; doi:10.1098/rsbl.2019.0485)
Supplement: Supplemental Figures [file rsbl20190485supp5.docx]

Susan E. Parks, Dana A. Cusano, Sofie M. Van Parijs, Douglas P. Nowacek (2019)

Acoustic crypsis in communication by North Atlantic right whale mother-calf pairs on the calving grounds. Published in Biology Letters

**Supplemental Figure 1.** A figure showing the distribution of call RL and background noise levels by individual ID. Lactating females are denoted with circles, other age/sex classes are denoted by squares.


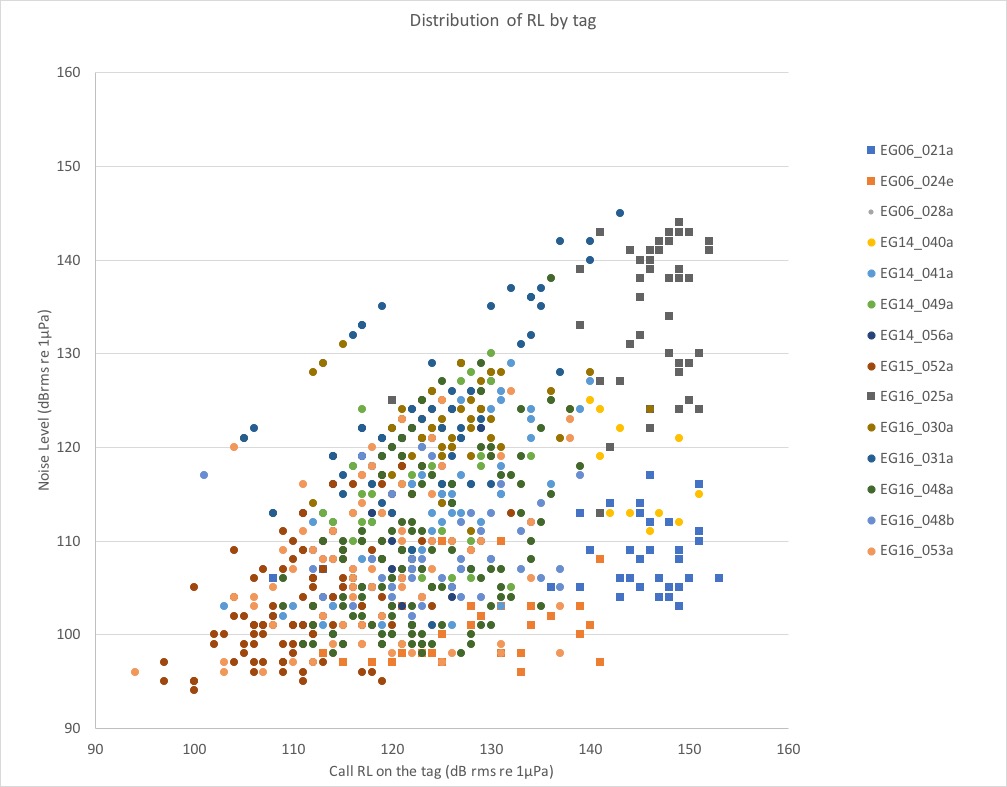


**Supplemental Figure 2.** A plot showing the k-means density plots by call RL for high and low amplitude clusters.
